# Preparation of NiO two-dimensional grainy films and their high-performance gas sensors for ammonia detection

**DOI:** 10.1186/s11671-015-0807-5

**Published:** 2015-03-11

**Authors:** Jian Wang, Pan Yang, Xiaowei Wei, Zhihua Zhou

**Affiliations:** School of Materials Science and Engineering, Xihua University, Chengdu, 610039 People’s Republic of China; State Key Laboratory of Electronic Thin Film and Integrated Devices, School of Microelectronics and Solid-state Electronics, University of Electronic Science and Technology of China, Chengdu, 610054 People’s Republic of China

**Keywords:** Nickel oxide films, Porous structure, Gas sensing device, Sensitivity and selectivity

## Abstract

Semiconductor NiO two-dimensional grainy films on glass substrates are shown to be an ammonia-sensing devices with excellent comprehensive performance, such as the good stability, short response time, outstanding recovery performance, excellent sensitivity, and selectivity. The morphology and structure analysis of gas sensing materials indicated that the as-fabricated NiO films was uniform and highly ordered porous structure on substrates, which composed of small size particles with diameters ranging from 8 to 30 nm. The shells of these particles were ultrathin amorphous NiO plates, and the core of each particle was face-centered cubic single crystal structure. In the gas sensing performance tests, we found that the excellent electron transport and interconnection properties of sensing films improved the stability and recovery performance of sensors, and porous surface structure increased the specific surface area of sensing films leading to fast response and excellent sensitivity for sensors. Meanwhile, this sensors owned outstanding selectivity toward ammonia which could be because NiO-sensing films had higher binding affinity for the electron-donating ammonia.

## Background

Design and fabrication of miniaturized gas sensing devices based on micro-, meso-, and nanostructure-sensing materials, with stable and controlled size and shape, has become a very advanced research topic because these novel portable devices are being widely applied in different fields of science and technology, such as homeland security, biological detection, and monitoring of manufacturing environments [1-5]. The central factors affecting the sensing performances of gas sensors depend not only on the structure, dimension, size, and morphology of sensing materials but also on their composition [6,7]. Therefore, nanostructure semiconductor metal oxides, such as CuO, SnO_2_, ZnO, NiO, TiO_2_, and WO_3_, have drawn great attention from a part of the scientific community to investigate various environmental monitoring issues [8-11]. Among these metal oxides, nickel oxide (NiO, band gap energy from 3.6 to 4.0 eV) nanomaterials, which are natural p-type semiconductors with high electron transport performance, have been recognized as the leading candidate for gas sensing devices due to their good sensitivity, low cost, and high compatibility with micromachining [5,12]. Currently, some miniaturized gas sensors with excellent sensitivity based on nanoscale NiO have been successfully fabricated, and even a few companies have offered this type of gas sensors in emerging markets [13,14]. However, further research is still necessary for further enhancing the sensing stability and continuing the expansion of the area of application of this type of sensor.

Over the past 10 years, tremendous efforts have been devoted to fabricating various morphologies of nanoscale NiO (such as NiO nanoparticles, nanotubes, nanowires, and nanorods) using assembling miniaturized sensors, and their gas sensing performance have been investigated by many scholars [15-18]. These sensors based on zero-dimensional or one-dimensional NiO which were assembled via placing the sensing materials on the special interdigital electrodes on various substrates, and these sensors had excellent sensitivity, fast response, and recovery; nevertheless, their stability was poor because the complicated manufacturing process leaded to the poor interconnection between the sensing materials and the electrodes [19]. In order to overcome the defects of this type of gas sensing devices, NiO nanoscale films, and foils which used assembling sensors have been prepared via different deposition techniques, such as metal evaporation, reactive sputtering, chemical vapor deposition, sol-gel, and chemical methods [20-24]. Among them, the chemical technique is a facile and controllable approach for preparing oxide films. For example, NiO films fabricated via chemical reduction have high porosity, uniform morphology, nanocrystallinity, and continuity, which are important for gas sensing performance, such as stability, repeatability, and sensitivity [5,25].

In the present work, we present a hydrothermal controllable approach at atmospheric pressure combined with high-temperature oxidation route in air to fabricate NiO films on the glass substrates and systematically investigate the gas sensing performance of the sensors based on as-prepared NiO films. The results showed that this type of sensor not only has outstanding stability and high sensitivity but also maintains excellent selectivity.

## Methods

### Chemicals and reagents

Analytical grade nickel chloride hexahydrate (NiCl_2_ · 6H_2_O) and hydrazine hydrate (N_2_H_4_ · 6H_2_O) were purchased from Sinopharm Chemical Reagent Co., Ltd., Shanghai, China. The sodium hydroxide (NaOH) and ethanol (C_2_H_5_OH) were purchased from Shanghai Chemical Reagents Company, Shanghai, China, and they were directly used without further purification. Distilled water was used to prepare all the solutions in our experiments, and ethanol and distilled water were used to rinse the samples and glass substrates.

### Preparation of the gas sensing materials

**Figure 1:**
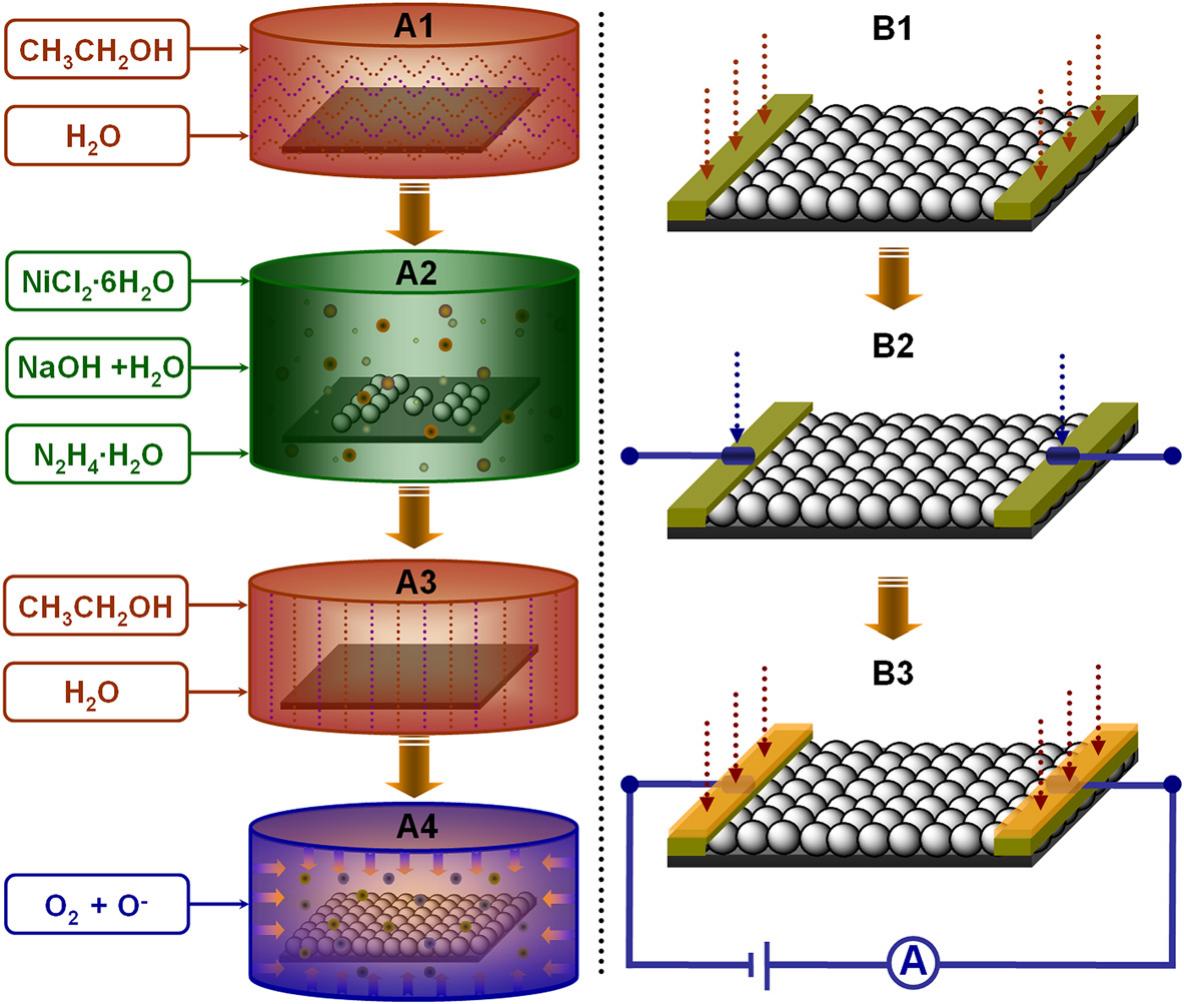
**Fabricating flowchart of the gas sensing materials and gas sensors.** **(A1-A4)** Flowchart of gas sensing films fabrication shows the specific procedures of synthesis of NiO films on glass substrates. This unique film structure was fabricated via a chemical controllable reaction combined with high-temperature oxidation approach. **(B1-B3)** Flowchart shows the assemble processes and configuration of the gas sensing devices based as-prepared NiO films, which mainly contained the preparation of electrodes and the connection between NiO sensing materials and electrodes.

The specific strategies to fabricate the NiO films on glass substrates, where the thickness of glass was around 2 mm, were summarized as follows. Initially, a certain amount of NiCl_2_ · 6H_2_O was dissolved into 50 mL distilled water and was continuous stirred at room temperature for 30 min to yield a grass-green transparent and homogeneously solution. Then, 50 mL aqueous solution of NaOH and 25 mL aqueous solution of N_2_H_4_ · 6H_2_O were added dropwise into the above as-prepared solution. This reaction mixture was stirred constantly for an hour to ensure that the anions and cation were dispersed homogeneously in the solution, and the color of solution changed from grass-green to navy blue color. In the meantime, the glass substrates were ultrasonically washed two times with ethanol and distilled water for 15 min, respectively. At this point, as-prepared reaction mixture was transferred into a flask containing as-treated glass substrate and subsequently heated to 80°C at atmospheric pressure. After the reaction was completed, the resulting Ni film covering of the glass substrate was washed two times using ethanol and distilled water, respectively, and then, the samples were heated to 450°C for 6 h in open air. The resultant gray-green products on the glass substrate were NiO films. Figure 1 A1-A4 shows the flowchart of fabrication of the NiO films on glass substrates, which used as gas sensing materials.

### Assembling of the gas sensors

The preparation procedures of the gas sensing devices were shown in Figure 1 B1-B3. Firstly, two sides of the glass substrate covering with NiO films were sputtered 20 nm pure titanium (Ti) and subsequently were connected with the positive and negative electrodes using the special silver paste, respectively. In order to ensure the excellent connection between gas sensing film and electrodes, the edges of glass substrates were strengthened via sputtering 130 nm pure gold (Au) in a magnetron sputtering apparatus. Secondly, two electrodes were bound to the gas sensing sockets using microscale Au wires in the miniaturized welding machine. Finally, the as-fabricated gas sensing devices were sonicated in ethanol and distilled washed, respectively, and then, vacuum heat treatment at 300°C for 2 h was executed in order to optimize the contact between NiO-sensing films and Au electrodes.

### Characterization of gas sensing materials

The surface microstructure and elemental composition of the NiO films were analyzed via a field emission scanning electron microscopy (FESEM) and energy-dispersive spectroscopy (EDS) at accelerating voltage of 5 and 20 kV, respectively. The structural performance of as-prepared gas sensing samples were investigated via X-ray powder diffraction (XRD) using an 18-kW advanced X-ray diffractometer in the two theta range from 30° to 90° with a Cu Kα radiation (λ = 0.154056 nm) rotating anode point source operating at 40 kV and 40 mA. In addition, high-resolution transmission electron microscopy (HRTEM) images were recorded in a JEM-2010 transmission electron microscope (JEOL Ltd., Akishima-shi, Japan) operating at 200 kV.

## Results and discussion

### Structure and morphology of gas sensing materials

**Figure 2:**
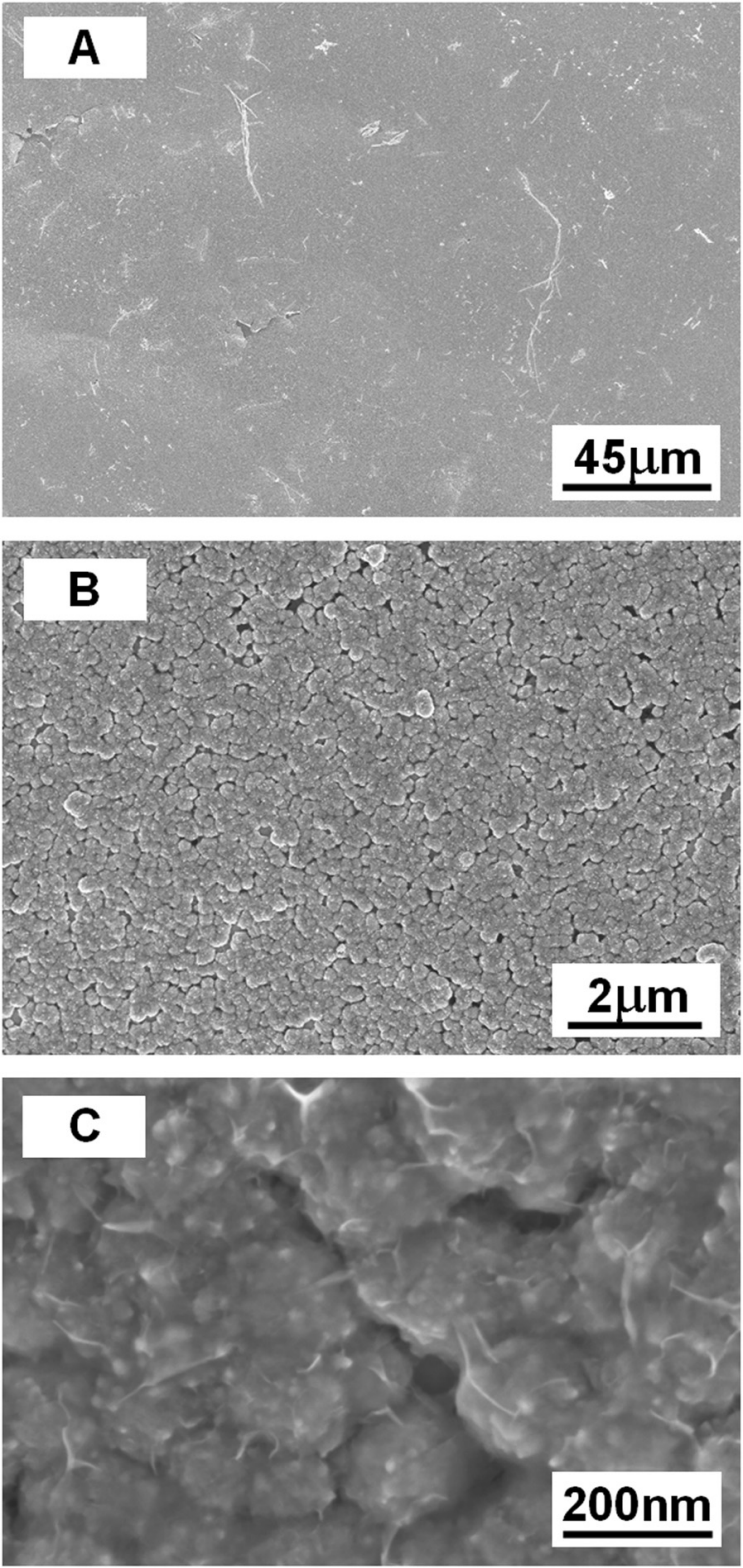
**SEM images of NiO film. (A)** Low-magnification, **(B)** medium-magnification, and **(C)** high-magnification SEM image demonstrating NiO particles distributing all over the glass surface forming NiO film.

In the first stage of the synthesis of NiO films, a controlled hydrazine hydrate reduction approach in aqueous solution has been developed to fabricate Ni films at atmospheric pressure. In the second stage, as-synthesized Ni films were gradually oxidized to NiO films at high temperatures in open air. Figure 2A presents the scanning electron microscopy (SEM) image of the as-prepared NiO films distributing all over the glass substrates. The morphology of the samples has been identified as two-dimensional grainy films, with an average of 300 nm film thickness. Figure 2B clearly shows that the NiO films had a uniform distribution of particles with a diameter of about 200 nm on the glass surface. In order to better understand the constitutes and structures of NiO films, further observation at higher magnification (Figure 2C) has revealed that these films are highly porous, and each particle composed of NiO film was assembled of small size particles with diameters ranging from 8 to 30 nm. This uniform and highly ordered porous morphology is very important for the application of gas sensing devices, and these small size particles provide large surface area which could adsorb more gas molecules to improve the sensitivity of sensors.

**Figure 3:**
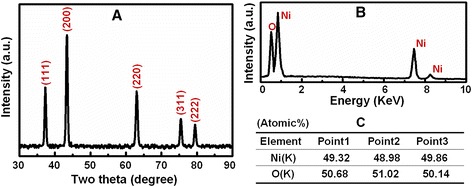
**XRD and EDS analysis of NiO film. (A)** XRD pattern of NiO film. **(B)** EDS analysis confirming that the as-prepared films are composed of only Ni and O elements. **(C)** EDS analysis at two different detection points of NiO film.

**Figure 4:**
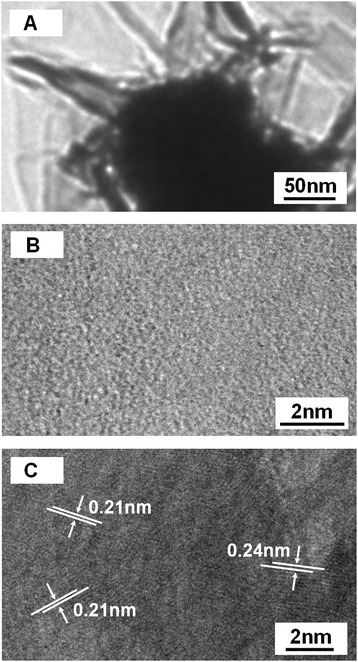
**TEM and HRTEM analysis of NiO film. (A)** TEM image of the small size particle-composed NiO film. **(B)** HRTEM image recorded from the shells of the small size particle. **(C)** HRTEM images recorded from the core of the small size particle.

Figure 3A shows the X-ray diffraction patterns of the as-fabricated films on the glass substrates. From the literatures (Joint Committee on Powder Diffraction Standards (JCPDS) card No. 47-1049), all the diffraction peaks of the films match well with the NiO face-centered cubic (fcc) structure. The peaks at scattering angles (two theta) of 37.48°, 43.47°, 62.97°, 75.62°, and 79.61° correspond to crystal planes of (111), (200), (220), (311), and (222) of crystalline NiO, respectively. From this XRD patterns, no other impurity peaks, such as nickel or nickel hydroxide, were detected, indicating that no other products existed in as-fabricated NiO films. EDS analysis (Figure 3B) of NiO film only revealed the peaks of Ni and O, and a similar atomic percentage of Ni and O was observed at two different detection points (Figure 3C), which further confirms that the as-synthesized NiO film are of high purity. In order to better understand the internal microstructure of the small size particle-composed NiO film in more detail, further transmission electron microscopy (TEM) observation is shown in Figure 4A, which shows that the shells of these particle was composed of many ultrathin plates. And then, we perform HRTEM characterization, as shown in Figure 4B, the fuzzier image (i.e., no any lattice fringes) which illustrates that these ultrathin plates were amorphous NiO structures. In the HRTEM image of the core of particle (Figure 4C), the clear lattice fringes indicate that the small size particle-composed NiO film is single crystal structure, and no visible line or planar defects imply the high crystallinity. This crystal is imaged to have nearly parallel lines, which are atomic planes separated via about 0.21 and 0.24 nm in Figure 4C, corresponding to both the {200} planes and the {111} planes of fcc NiO crystal, respectively.

### Sensing performance of the gas sensor

Ammonia sensors based on NiO films on glass substrates were fabricated via connecting the gas sensing materials to the positive and negative electrodes using the special silver paste combined with subsequent sputtering pure Au, as shown in Figure 1 B1-B3. In our experiments, the conductance between electrodes was measured with a precision semiconductor parameter analyzer to investigate the ammonia-sensing performance. The response and recovery time of gas sensing devices are defined as the time needed to reach 95% of the final steady state conductance upon exposure to the testing gas [26].

**Figure 5:**
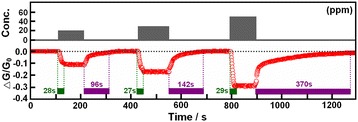
**Stability and sensitivity test of gas sensors based on NiO films.** The conductance change of NiO films on the glass substrates to 20, 30, and 50 ppm of NH_3_ at room temperature.

Figure 5 shows the dynamic sensing transients of gas sensors based on NiO films to 20, 30, and 50 ppm NH_3_. In the sensing transients, the gas sensor exhibits the stable sensitive signals, which because the two-dimensional grainy films as a whole was able to quickly and stably transfer electronics between the gas molecules and the gas sensing materials [7]. In three sensing cycle experiments, we found that the NiO film sensor owned the fast response and excellent sensitivity. For example, when the sensor was exposed to 30 ppm NH_3_ for about 27 s (short response time) without the need for pre-concentration step, the conductance change reached up to about 18%, which because the large specific surface area (porous surface structure) of NiO film provided good accessibility of the gas molecules to the sensing materials. Meanwhile, it is obvious that the conductance change increases with the increasing NH_3_ concentration, and more importantly, we find that that after three sensing cycle tests, it takes less than 7 min to completely recover via blowing the air combined with illumination using an infrared lamp, which is important for the gas sensing devices applications. This excellent recovery performance is due to the good interconnection between NiO film-sensing materials and two electrodes.

**Figure 6:**
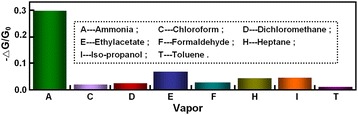
**Selectivity tests of gas sensors based on NiO films.** The concentration of NH_3_ is 50 ppm, and the concentration of other organic gases is higher than 300 ppm.

Selectivity of the gas sensor is one of the most important gas sensing performances, which relies mainly on the specific interactions between gas sensing materials and target gas molecules [27]. The gas sensing performance of sensor based on NiO films toward a variety of toxic, corrosive, and flammable gases including ammonia, chloroform, dichloromethane, ethylacetate, formaldehyde, heptane, iso-propanol, and toluene were tested to evaluate the selectivity of sensor, as shown in Figure 6. Remarkably, this gas sensor exhibited excellent selectivity to NH_3_ when exposed to these interfering gases. For instance, the conductance change of the sensors upon exposure to 50 ppm of NH_3_ was about 30%, whereas that the change in conductance was less than 8% to higher concentration (greater than 300 ppm) of other gases. These results suggest that the assembling porous NiO films on glass substrates is an excellent strategy for simultaneously promoting the stability, sensitivity, and sensitivity of the gas sensing devices.

## Conclusions

In summary, semiconductor NiO gas sensing devices were successfully fabricated using NiO film prepared via a chemical reaction combined with subsequent high-temperature oxidation method. NiO films composed of nanoscale crystallites with particle diameters ranging from 8 to 30 nm, and the morphology of this film was uniform and highly ordered porous which was important for simultaneously promoting the stability, sensitivity, and sensitivity of the gas sensing devices. Significantly, the gas sensors based on as-fabricated NiO grainy films exhibits the stable sensitive signals, short response time, outstanding recovery performance, excellent sensitivity, and selectivity toward ammonia over other organic gases, such as chloroform, dichloromethane, ethylacetate, formaldehyde, heptane, iso-propanol, and toluene. It is suggested that the method we demonstrated here could also be extended to other two-dimensional grainy films for corresponding gas sensing applications.
